# Clinical characteristics of Stevens-Johnson syndrome/toxic epidermal necrolysis-like reactions induced by immune checkpoint inhibitors

**DOI:** 10.1093/oncolo/oyaf143

**Published:** 2025-06-10

**Authors:** Ziliang Zheng, Zhu Shen

**Affiliations:** Guangdong Cardiovascular Institute, Guangdong Provincial People’s Hospital, Guangdong Academy of Medical Sciences, Guangzhou 510080, China; Department of Dermatology, Guangdong Provincial People’s Hospital (Guangdong Academy of Medical Sciences), Southern Medical University, Guangzhou 510080, China; Guangdong Cardiovascular Institute, Guangdong Provincial People’s Hospital, Guangdong Academy of Medical Sciences, Guangzhou 510080, China; Department of Dermatology, Guangdong Provincial People’s Hospital (Guangdong Academy of Medical Sciences), Southern Medical University, Guangzhou 510080, China

**Keywords:** immune checkpoint inhibitors, immune-related adverse events, severe cutaneous adverse reactions, Stevens-Johnson syndrome, toxic epidermal necrolysis

## Abstract

**Background:**

Immune checkpoint inhibitors (ICIs) have demonstrated significant therapeutic benefits but are also associated with skin-related adverse reactions. The specific characteristics of severe adverse reactions caused by ICIs remain unclear.

**Objective:**

To investigate the disease characteristics of Stevens-Johnson syndrome (SJS)/toxic epidermal necrolysis (TEN)-like reactions induced by ICIs.

**Methods:**

Cases of ICI-induced SJS/TEN were collected from PubMed, CNKI, Wanfang Data Knowledge Service Platform, and Guangdong Provincial People’s Hospital, with a search time span ranging from March 2011 to January 31, 2024.

**Results:**

A total of 110 cases of SJS, TEN, and overlapping SJS/TEN were analyzed, with a male predominance (62%). Mucous membrane involvement was observed in 71 patients (66%), though less frequently than in classic SJS/TEN. The mean latency period was 64 days, varying by subtype (105 days for SJS and 53 days for TEN). Combination therapy with ICIs was associated with a higher mortality risk (*P* = .029). Deceased patients exhibited shorter latency periods (mean 30.3 days) and more severe mucosal involvement (up to 100%), although the differences were not statistically significant. Systemic glucocorticoid therapy was the cornerstone of treatment for SJS/TEN-like reactions. The addition of immunoglobulin showed a trend toward improved outcomes but did not significantly affect mortality or cure rates compared to glucocorticoid monotherapy. The combination of systemic glucocorticoids and antibiotics demonstrated a promising trend, with a higher proportion of patients in the improvement/cure group using this regimen (*P* = .085).

**Conclusions:**

This study summarizes the clinical characteristics of ICI-induced SJS/TEN-like reactions, providing insights into their features and potential treatment strategies for severe skin-related adverse events induced by ICIs.

**Implications for Practice**

This study highlights the clinical characteristics and management strategies for SJS and TEN-like reactions induced by ICIs. Key findings include a higher mortality risk with combination ICIs therapy, shorter latency periods in fatal cases, and the importance of systemic glucocorticoids as a cornerstone treatment. The addition of antibiotics showed promising trends in improving outcomes; however, definitive conclusions cannot be drawn due to limited statistical power. These observations highlight the importance of infection control in severe irAEs and call for further investigation into antibiotic timing, selection, and patient stratification. These insights emphasize the need for early detection, close monitoring of mucosal involvement, and timely intervention to reduce mortality. This research provides valuable guidance for clinicians managing severe skin-related adverse events in cancer patients receiving ICIs.

## Introduction

Immune checkpoint inhibitors (ICIs) are a class of therapeutic agents that function by blocking specific proteins on immune cells, known as immune checkpoints. These checkpoints normally regulate the immune system to prevent excessive immune responses and autoimmunity. However, cancer cells can exploit these checkpoints to evade immune surveillance. By targeting these proteins, ICIs disable their inhibitory effects, enabling the immune system to recognize and attack cancer cells more effectively. Examples of such inhibitors include drugs targeting CTLA-4, PD-1, and PD-L1 proteins.^1^ Although ICIs have significantly improved cancer treatment, they are associated with immune-related adverse events (irAEs). Among these, Stevens-Johnson syndrome (SJS)/toxic epidermal necrolysis (TEN)-like irAEs are rare but severe skin reactions that can be life-threatening. Stevens-Johnson syndrome and TEN are severe mucocutaneous reactions with epidermal detachment and mucosal erosions. Classification is based on affected body surface area (BSA): SJS < 10% BSA, SJS/TEN overlap 10%-30% BSA, TEN > 30% BSA. Diagnosis involves considering patient factors, clinical manifestations, and histopathological findings.

Classical SJS/TEN is linked to specific HLA types and triggered by drugs like carbamazepine and allopurinol. Its pathogenesis involves cytotoxic T cell-mediated keratinocyte apoptosis via Fas-FasL and perforin/granzyme pathways. In contrast, ICI-induced SJS/TEN-like reactions stem from sustained immune activation (eg, PD-1 blockade-driven CD8 + T cell hyperactivity), similarly disrupting keratinocytes. Systemic glucocorticoids likely suppress these activated immune cells. Clinically, both conditions manifest as painful erythematous rashes, blisters, and erosions (face/trunk to extremities), with mucosal involvement (eyes, mouth, genitalia) and Nikolsky sign. Treatment includes stopping the causative agent, supportive care (hydration, analgesia, wound care), glucocorticoids, and occasionally Intravenous Immunoglobulin (IVIG).^2^ In this study, our aim is not to directly compare with classical SJS/TEN cases, but rather to focus on exploring the unique characteristics and management strategies of SJS/TEN-like reactions induced by ICIs.

The clinical features and optimal management strategies for these adverse reactions remain poorly understood. Furthermore, the immunological mechanisms or risk factors that are still unknown contribute to the complexity of managing these conditions. Therefore, this study aims to analyze the clinical characteristics of SJS and TEN-like reactions induced by ICIs, thereby enhancing understanding of these severe adverse events.

## Methods

### Patients

In this study, cases of ICI-induced SJS/TEN-like irAEs were collected from PubMed, CNKI, Wanfang Data Knowledge Service Platform, and Guangdong Provincial People’s Hospital. To avoid duplicate case inclusion across databases, a rigorous deduplication process was implemented. Patient characteristics, including age, gender, tumor type, and ICI administration timeline, were cross-checked among all identified cases. Cases with identical or highly overlapping demographic and clinical profiles were consolidated into a single entry. The inclusion of both English and Chinese literature, alongside real-world clinical data, ensured broad geographic and linguistic coverage, thereby minimizing regional bias.^3^

The search terms included: (((Stevens-Johnson Syndrome) OR SJS) OR ((Toxic Epidermal Necrolysis) OR TEN)) AND (((Immune Checkpoint Inhibitors) OR ICI) OR pembrolizumab OR nivolumab OR ipilimumab OR sintilimab OR carilizumab OR tislelizumab OR triprolizumab OR ((immune-related adverse events) OR (irAEs)) OR (PD-1 OR PD-L1 OR CTLA-4 OR CTLA4)). The search period was from March 2011 (the release date of the first ICI inhibitor, Ipilimumab) to January 2024. The inclusion criteria were cases that adopted the internationally agreed diagnostic criteria for SJS/TEN (including typical clinical manifestations, ICI medication history, skin biopsy results, and SCORTEN score). Cases of SJS/TEN-like irAEs unrelated to ICI treatment and literature published in languages other than English were excluded. To ensure precision and relevance, we adopted a rigorous case selection strategy. First, cases unrelated to ICI treatment were excluded, ensuring all included cases were ICI-induced. Second, cases involving PD-1 inhibitors alone but leading to non-SJS/TEN immune-related adverse events were also excluded, maintaining the study’s specificity and accuracy.

The collected data included patients’ demographic and clinical information, tumor types, antitumor treatments, comorbidities, SJS/TEN presentations, laboratory findings, skin biopsy characteristics, involvement of other organ systems, treatment strategies, SJS/TEN outcomes, and subsequent antitumor treatments. This study was approved by the Medical Ethics Committee of Guangdong Provincial People’s Hospital (approval number: KY2024-588-02).

### Statistics

The categorical variables were represented by crosstabs and analyzed statistically by chi-square test. Continuous variables were represented by mean ± standard deviation and analyzed by descriptive statistics and hypothesis testing statistics, including *t*-test for comparisons between groups when the data met the assumptions of normality and equal variance. Statistical significance analysis was defined by whether the *P*-value was less than .05. In this study, all statistical analyses were performed using SPSS software (version 19.0, SPSS Inc., Chicago, IL, USA).

## Results

### Male predominance and lung cancer prevalence in ICIs-induced SJS/TEN cases

A total of 217 cases were retrieved, of which 110 met the criteria, including 83 from PubMed (https://pubmed.ncbi.nlm.nih.gov/), 25 from CNKI (https://www.cnki.net/) and Wanfang databases (https://www.wanfangdata.com.cn/), and 2 from Guangdong Provincial People’s Hospital, comprising 68 males (62%) and 42 females (38%). The mean age of onset was 63 ± 13 years, indicating that most patients were elderly. Male patients outnumbered females across the overall group, as well as in the SJS, SJS/TEN, and TEN subgroups (Table 1; Table S1).

**Table 1.** Demographics and clinical characteristics of ICI-induced SJS/TEN-like irAEs.

	No. (%)			
	Total	SJS-like	SJS/TEN-like	TEN-like
	110	33	9	68
**Gender**				
Male	68 (62%)	19 (58%)	6 (67%)	43 (63%)
Female	42 (38%)	14 (42%)	3 (33%)	25 (37%)
**Age of onset (years)**	63 ± 13	64 ± 14	58 ± 17	63 ± 12
**Tumor diagnosis to ICIs initiation (months)**	21 ± 43	20 ± 51	33 ± 61	16 ± 27
**Latency period (days)**	64 ± 134	105 ± 210	13 ± 72	53 ± 91
**Mucosal involvement**				
Yes	71 (66%)	29 (88%)	8 (89%)	34 (50%)
No	3 (3%)	0	0	3 (44%)
**Total treatment days (mean days)**	35 ± 20	43 ± 44	19 ± 13	32 ± 23
**Prognosis**				
Recovery	37 (34%)	13 (39%)	3 (33%)	21 (31%)
Improvement	41 (37%)	16 (48%)	5 (56%)	20 (29%)
No Effect	1 (1%)	0	0	1 (1%)
Death	23 (21%)	2 (6%)	1 (11%)	20 (29%)
**Glucocorticoid therapy (recovery group)**				
Mean days of treatment	36.10 ± 20.53	43.80 ± 23.28	20 ± 13.23	34.5 ± 18.78
Mean glucocorticoid dose (mg, in prednisone)	2389 ± 1447	2639 ± 2035	1200 ± 990	2414 ± 1100
**Combined immunoglobulin and death outcome**				
Glucocorticoid *plus* immunoglobulin	57/14 (25%)	8/1 (13%)	5/1 (20%)	44/12 (27%)
Glucocorticoid *without* immunoglobulin	44/5 (11%)	21/1 (5%)	3/0	20/4 (20%)
**Combined immunoglobulin and recovery outcome**				
Glucocorticoid *plus* immunoglobulin	57/17 (30%)	8/3 (38%)	5/1 (20%)	44/13 (30%)
Glucocorticoid *without* immunoglobulin	44/19 (43%)	21/9 (43%)	3/2 (67%)	20/8 (40%)
**Combined biologics and death outcome**				
Glucocorticoid *plus* biologics	11/2 (18%)	3/0	0	8/2 (25%)
Glucocorticoid *without* biologics	90/17 (19%)	26/2 (8%)	8/1 (13%)	56/14 (25%)
**Combined biologics and recovery outcome**				
Glucocorticoid *plus* biologics	11/5 (45%)	3/2 (67%)	0	8/3 (38%)
Glucocorticoid *without* biologics	90/31 (34%)	26/10 (38%)	8/3 (38%)	56/18 (32%)

Abbreviations: ICI, immune checkpoint inhibitors; SJS, Stevens-Johnson syndrome; TEN, toxic epidermal necrolysis; irAEs, immune-related adverse events.

Among ICIs-induced SJS/TEN cases, lung cancer was the most common, accounting for 36 cases (33%). Regarding tumor types, squamous cell carcinoma (27 cases, 25%), melanoma (21 cases, 19%), and adenocarcinoma (17 cases, 15%) were predominant (Table 1; Table S1).

### Prolonged latency and mucosal involvement in SJS/TEN-like irAEs

Compared to classic drug-induced allergic reactions, the average latency period of SJS/TEN-like irAEs was longer, averaging 64 ± 134 days. Among these, the latency for TEN (53 days) was shorter than for SJS (105 days). Although this difference was not statistically significant (*P* = .114), it suggests that TEN may progress more rapidly (Table 1; Table S1). Additionally, a considerable proportion of patients (66%) experienced mucosal involvement, such as in the eyes and lips. However, this percentage was lower than that typically observed in classic SJS/TEN cases (Table 1; Table S1).

### Analysis of monotherapy and combination therapy with systemic glucocorticoids

Overall, the recovery rate of SJS/TEN-like irAES was 34%, with an improvement rate of 37% and a mortality rate of 21%. Notably, the mortality rate for SJS-like irAE was 6%, while TEN-like had a much higher mortality rate of 29% (Table 1; Table S1).

In terms of systemic treatment, the mean duration of systemic glucocorticoid therapy in the recovery group was 36.10 ± 20.53 days, with a mean cumulative dosage of 2389 ± 1447 mg (prednisone equivalent). For systemic glucocorticoid combined with immunoglobulin therapy, the mortality rate in the combination group (25%) was higher than that in the glucocorticoid-only group (11%). This can be explained by the fact that patients requiring combination therapy typically had more severe conditions. Similarly, the recovery rate in the combination group (30%) was lower than in the glucocorticoid-only group (43%) (Table 1; Table S1).

For systemic glucocorticoid combined with biologics, while the recovery rate appeared higher, the difference did not reach statistical significance (*P* = .472, Table 1; Table S1). This trend may indicate a synergistic effect of biologics with glucocorticoids, but confirmation through multivariate analysis in larger datasets is imperative. In the cases of systemic glucocorticoid combined with plasma exchange or antibiotics, combined therapies did not consistently reduce mortality or improve recovery rates significantly. However, timely use of antibiotics played a role in reducing infections and improving prognosis. Specifically, the addition of antibiotics increased the recovery rate from 33% to 41%, particularly in TEN cases where the recovery rate rose from 24% to 48% (p = 0.055, Table 1; Table S1).

### Use of combined ICIs in death cases of severe SJS/TEN-like irAEs

Next, the SJS/TEN and TEN patients were categorized into 2 outcome-based groups: Recovery & Improvement (49 cases) and Death (21 cases, Table 2; Table S2). Males were predominant in both groups, and the gender distribution showed no significant difference (*P* = .244, Table 2; Table S2). Regarding tumor type, melanoma had the highest proportion in the death group (48%), indicating that melanoma patients should be particularly cautious about fatal SJS/TEN-like reactions when using ICIs (*P* = .003, Table 2; Table S2). In terms of ICI usage, most patients received PD-1 or PD-L1 inhibitors, while the combination of PD-1 and CTLA-4 inhibitors was more frequent in the death group (29%), showing statistical significance (*P* = .029, Table 2; Table S2). This suggests that combined ICI therapy may increase the risk of mortality.

**Table 2.** Analysis of demographic and clinical features in fatal vs. recovered/improved cases of severe ICI-induced SJS/TEN-like irAEs.

SJS/TEN &TEN-like irAEs	*N* (%)	
	Recovery and improvement	Death
**Total**	49	21
**Gender**		
Male	35 (71%)	12 (57%)
Female	14 (29%)	9 (43%)
**Severity**		
SJS/TEN-like	8 (16%)	1 (5%)
TEN-like	41 (84%)	20 (95%)
**ICIs usage**		
PD-1 and PD-L1 inhibitors^a^	46 (94%)	15 (71%)
Others (PD-1 plus CTLA-4 inhibitors)	3 (6%)	6 (29%)
**Latency period (days)**	55.77 ± 102.47	30.31 ± 30.31
**Mucosal involvement**		
Yes	30 (91%)	8 (100%)
No	3 (9%)	0
**Treatment with systemic glucocorticoids**		
Apply	48 (98%)	17 (81%)
Not Applied	1 (2%)	4 (19%)
Average treatment days	32.12 ± 18.41	34 ± 32.52
Mean glucocorticoid dose (mg, in prednisone)	2247.92 ± 1578.05	2526.25 ± 2018.43
**Systemic glucocorticoid *plus* immunoglobulin**		
Apply	32 (67%)	13 (62%)
Not Applied	16 (33%)	8 (38%)
Average treatment days	30.67 ± 14.24	35.3 ± 35.30
Mean glucocorticoid dose (mg, in prednisone)	1938.56 ± 1250.69	2526.5 ± 2120.36
**Systemic glucocorticoid plus antibiotic therapy**		
Apply	22 (46%)	5 (24%)
Not Applied	26 (54%)	16 (76%)
Average treatment days	34.63 ± 24.89	50 ± 47.61
Mean glucocorticoid dose (mg, in prednisone)	2110.63 ± 1348.32	3030 ± 1065.22
**Total treatment days (mean days)**	31.48 ± 18.66	32 ± 31.96
**Total glucocorticoid dose (mg, in prednisone)**	2247.92 ± 1578.05	2526.25 ± 2018.43

^a^There was only one death with PD-L1 inhibitors (atezolizumab).

Abbreviations: ICI, immune checkpoint inhibitors; SJS, Stevens-Johnson syndrome; TEN, toxic epidermal necrolysis; irAEs, immune-related adverse events.

### Shorter latency and greater mucosal involvement in death SJS/TEN and TEN cases

The latency period in the death group was relatively shorter, averaging 30.3 days compared to 55.8 days in the recovery/improvement group. Although this difference did not reach statistical significance (*P* = .334, Table 2; Table S2), the trend toward shorter latency in fatal cases underscores the importance of early recognition and intervention. Future studies with expanded sample sizes should explore the prognostic value of latent periods. Regarding mucosal involvement, all patients in the death group had mucosal sites affected (100%, *P* = 0.897, Table 2; Table S2). While the difference was not statistically significant, this finding highlights the need for close attention to mucosal involvement in clinical practice.

### Systemic glucocorticoid therapy as a key intervention for fatal severe SJS/TEN-like irAEs

Systemic glucocorticoid therapy was used more frequently in the improvement/recovery group (*P* = .043, Table 2; Table S2), underscoring its importance in treating life-threatening SJS/TEN and TEN-like reactions. Although the proportions of patients receiving systemic glucocorticoids combined with immunoglobulin or antibiotics varied between the 2 groups, these differences were not statistically significant and warrant further investigation with larger sample sizes.

## Discussion

This study found that the average treatment duration for life-threatening SJS/TEN-like lesion was 35 days, providing key insights into the disease’s treatment timeline for better planning. TEN-like irAE has a 60% cure/improvement rate and a 29% mortality rate, reflecting its greater severity compared to SJS. These findings emphasize the need for more effective strategies to reduce TEN-like irAE mortality.

The study found that patients using combination ICIs had a higher risk of death, highlighting the need for early detection of SJS/TEN-like AEs through close monitoring of skin changes. However, literature reports indicate that factors including ipilimumab combination showed no significant risk among 67 patients with SJS/TEN treated with PD-1/PD-L1 inhibitors.^4^ The discrepancy in conclusions could potentially be attributed to differences in patient populations, sample sizes, and specific treatment regimens employed in the studies.

The latency period for life-threatening irAEs in this study (mean 64 days, from ICI initiation to irAE diagnosis) aligns with previously published data,^1,4-6^ significantly exceeding that of classic SJS/TEN drug eruptions. This prolonged latency can be attributed to the fundamentally different mechanisms underlying these conditions. Classic SJS/TEN is typically triggered by direct drug reactions, resulting in rapid onset. In contrast, irAEs caused by ICIs involve a gradual disruption of immune homeostasis, progressing from quantitative to qualitative changes over time. Furthermore, the long half-life of macromolecular PD-1/PD-L1 inhibitors means that steady-state concentrations are only achieved after several months.^1,4^ These factors collectively contribute to the extended latency period of SJS/TEN-like irAEs observed in ICI-treated patients.

Notably, our study found that the latency period for TEN-like irAEs (mean 53 days) was significantly shorter than for SJS-like irAEs (mean 105 days), indicating more rapid onset in TEN. This underscores the need for earlier intervention in TEN cases. Moreover, the latency period in the death group was even shorter, averaging 30.3 days. Although this difference was not statistically significant, likely due to the small sample size, the trend suggests faster disease progression in fatal cases. Early detection and timely treatment are essential to mitigate progression and reduce mortality. Larger studies are needed to validate these findings.

In classic SJS/TEN, mucosal involvement occurs in most patients, with up to 80% of cases affecting 2 or more mucosal sites. Oral involvement is the most common, presenting as mucositis and ulcers in nearly 100% of cases.^7^ However, in this study, mucosal involvement in ICI-induced SJS/TEN-like reactions was less frequent, possibly reflecting differences in pathological mechanisms between ICI-induced and classic SJS/TEN. Notably, patients in the death group exhibited more severe mucosal involvement, with 100% affected. These findings highlight the importance of monitoring mucosal involvement in SJS/TEN-like irAE patients.

It should be noted that the prognostic subgroup analyses were observational in nature and lacked randomization; consequently, the results may have been influenced by unmeasured confounders. This study showed that patients treated with systemic glucocorticoids had higher improvement/recovery rates and lower mortality (*P* = .043), underscoring their importance in SJS/TEN management. Systemic glucocorticoids exert potent anti-inflammatory and immunosuppressive effects, rapidly controlling disease progression and alleviating symptoms. Early identification and high-dose glucocorticoid therapy have been suggested as key factors for successful treatment.^8^ Notably, the shorter duration and lower cumulative glucocorticoid dosage in the recovery/improvement group suggest faster clinical responses, as quicker recovery may necessitate shorter treatment. Conversely, prolonged glucocorticoid use in fatal cases may indicate refractory disease or delayed intervention.

Although systemic glucocorticoids are effective in treating ICI-induced SJS/TEN-like reactions, long-term use poses risks. Infections, including bacterial, fungal, and viral, are common and serious complications due to immunosuppression. Additionally, metabolic disorders such as elevated blood sugar, high blood pressure, and osteoporosis may occur, especially in elderly patients.

In the treatment of ICI-induced SJS/TEN-like reactions, balancing efficacy and safety is crucial. Clinicians should aim to reduce glucocorticoid doses early to mitigate adverse effects while maintaining disease control. Close monitoring for infection, blood sugar, blood pressure, and bone health changes is essential for timely treatment adjustments and complication prevention. Individualized medication strategies, tailored to patient conditions, are necessary to optimize treatment outcomes while minimizing side effects. This study compared the recovery rates between the systemic glucocorticoids plus immunoglobulin group and the systemic glucocorticoids alone group, finding no significant difference. The recovery rate was 30% in the combined group and 43% in the glucocorticoid-only group, likely reflecting greater disease severity in the combination group. However, subtype analysis showed differences in recovery rates across SJS, SJS/TEN overlap, and TEN cases. Combination therapy achieved a 38% recovery rate in SJS compared to 43% with monotherapy. In contrast, the SJS/TEN overlap subgroup had a lower recovery rate with combination therapy (20%) versus monotherapy (67%). For TEN cases, combination therapy yielded a 30% recovery rate, while monotherapy provided a 40% recovery rate. These variations suggest differential therapeutic responses across subtypes, though statistical significance was limited due to small sample sizes and potential confounding factors such as disease severity. Literature suggests that immunoglobulin doses exceeding 2 g/kg may reduce mortality in TEN in 6 out of 8 studies, though potential biases exist.^9^ Additionally, in the improvement/recovery group treated with glucocorticoids plus immunoglobulin, patients required fewer glucocorticoids and shorter treatment durations. While not statistically significant, these trends may inform clinical practice. Larger studies with comprehensive clinical data are needed for further validation.

Patients treated with systemic glucocorticoids plus biologics had a higher recovery rate(45%) than those on glucocorticoids alone(34%), though this difference was not statistically significant (*P* = .472). Reports show that combining adalimumab with systemic glucocorticoids can lower mortality compared to glucocorticoid monotherapy.^10^ Notably, combined biologics appeared to reduce treatment duration for severe TEN-like AE from 35 to 23 days, although this difference also lacked statistical significance (*P* = .374). Further confirmation through multivariate analysis of large-scale data with complete clinical information is needed.

In the improvement/recovery group, the combined use of systemic glucocorticoids and antibiotics was more frequent, treatment duration shorter, and cumulative glucocorticoid use lower. These findings highlight the importance of early identification of infections and antibiotic use in SJS/TEN-like irAE management. However, the possibility that the improvement/recovery group had milder conditions cannot be excluded, necessitating further studies with larger sample sizes for validation. This study also found a lower mortality rate in the systemic glucocorticoids plus antibiotics group (17%) compared to glucocorticoids alone (29%). Although not statistically significant, likely due to limited sample size, this suggests the importance of timely antibiotic use, particularly as infections often occur in severe cases. Additionally, systemic glucocorticoids plus antibiotics increased recovery rates from 33% to 41%, especially in severe TEN cases (24% to 48%, *P* = .055). These results further support the timely use of antibiotics in SJS/TEN-like irAE treatment, warranting larger studies for confirmation.

While this study offers valuable insights, its findings are preliminary and limited by the sample size. Observed trends, including higher mortality risk with combination ICIs, shorter latency periods in fatal cases, and the effectiveness of systemic glucocorticoids, require validation through larger and more diverse prospective studies to reduce retrospective biases and provide more definitive conclusions.

In evaluating SJS/TEN-like reactions induced by ICIs, several confounding factors must be considered: neoplasm type, patient’s functional status, prior therapies, and treatment preferences. Considering these variables are essential for accurate clinical decision-making, study interpretation based on larger samples with multifactor analysis is required.

### Limitation

While this study offers valuable insights into the characteristics and treatment outcomes of ICI-induced SJS/TEN-like responses, it has some limitations. First, although the sample size was sufficient for preliminary analysis, it was relatively small for multivariable analysis and may not fully represent all affected patient populations. Specifically, certain combination therapy subgroups, such as ipilimumab plus nivolumab in the death group (*n* = 6, Table S2), had notably limited sample sizes. These findings should be interpreted with caution and require validation through larger-scale studies. Second, the retrospective design may introduce biases in data collection, processing, and interpretation. Future prospective studies with larger and more diverse cohorts are needed to validate and refine these findings.

## Conclusion

In conclusion, this study provides key insights into ICI-induced SJS/TEN-like AEs, showing a higher incidence in men and a 21% overall mortality rate, particularly elevated in patients on combination ICIs. Mucosal involvement, though less frequent than in classic SJS/TEN, indicates severe disease, with TEN-like reactions progressing faster than SJS-like ones. The death group had a shorter latency period compared to the improvement/recovery group. Systemic glucocorticoids remain the cornerstone of treatment, achieving high improvement/recovery rates. While further large-scale studies are needed to confirm the benefits of adding immunoglobulins, combining systemic glucocorticoids with antibiotics has shown promising trends in improving outcomes. These findings advance understanding of ICIs-induced severe skin AEs and lay the foundation for future research to refine clinical management.

## Supplementary Material

oyaf143_suppl_Supplementary_Tables_S1

oyaf143_suppl_Supplementary_Tables_S2

## Data Availability

The data underlying this article will be shared on reasonable request to the corresponding author. The data underlying this article are available in the article and in its Supplementary Material.
