# Supplementary material for: Clinical characteristics of Stevens-Johnson syndrome/toxic epidermal necrolysis-like reactions induced by immune checkpoint inhibitors
Source: Oncologist. 2025 Jun 10;30(6):oyaf143. doi: 10.1093/oncolo/oyaf143 (PMC12149091; doi:10.1093/oncolo/oyaf143)
Supplement: oyaf143_suppl_Supplementary_Tables_S1 [file oyaf143_suppl_supplementary_tables_s1.docx]

**Supplemental Table S1: Summary of extensive epidermal necrotic reactions based on diagnostic stratification**

|  | No. (%) | | | |
| --- | --- | --- | --- | --- |
|  | Total | SJS-like | SJS/TEN-like | TEN-like |
|  | 110 | 33 | 9 | 68 |
| **Gender** |  |  |  |  |
| Male | 68 (62%) | 19 (58%) | 6 (67%) | 43 (63%) |
| Female | 42 (38%) | 14 (42%) | 3 (33%) | 25 (37%) |
| **Mean age of onset (years)** | 63±13 | 64±14 | 58±17 | 63±12 |
| **Tumor site** |  |  |  |  |
| Lung | 36 (33%) | 16 (48%) | 3 (33%) | 17 (25%) |
| Liver | 10 (9%) | 1 (3%) | 0 | 9 (13%) |
| Stomach | 8 (7%) | 1 (3%) | 1 (11%) | 6 (9%) |
| Esophagus | 8 (7%) | 1 (3%) | 0 | 7 (10%) |
| Others ^a^ | 29 (26%) | 10 (30%) | 5 (56%) | 14 (21%) |
| Unmarked | 19 | - | - | - |
| **Tumor type** |  |  |  |  |
| Squamous carcinoma | 27 (25%) | 10 (30%) | 3 (33%) | 14 (21%) |
| Melanoma | 21 (19%) | 3 (9%) | 0 | 18 (26%) |
| Adenocarcinoma | 17 (15%) | 6 (18%) | 3 (33%) | 8 (12%) |
| Non-small cell carcinoma | 8 (7%) | 4 (12%) | 0 | 4 (6%) |
| lymphoma | 4 (4%) | 0 | 1 (3%) | 3 (4%) |
| Others ^b^ | 33 (30%) | 10 (30%) | 2 (22%) | 21 (31%) |
| **Tumor discovery - Application of ICIs duration (mean month)** | 21±43 | 20±51 | 33±61 | 16±27 |
| **ICIs usage** |  |  |  |  |
| PD-1 | 94 | 29 | 9 | 56 |
| PD - 1 + CTLA - 4 | 11 | 1 | 0 | 10! |
| PD-L1 | 2 | 1 | 0 | 1 |
| CTLA-4 | 0 | 0 | 0 | 0 |
| PD - L1 + CTLA4 | 0 | 0 | 0 | 0 |
| Not mentioned | 3 | 2 | 0 | 1 |
| **Incubation period (mean days)** | 64±134 | 105±210 | 13±72 | 53±91 |
| **With the pathological results** | 50 (45%) | 21 (64%) | 4 (44%) | 25 (37%) |
| **Involves special parts such as eyes, lips, etc.** |  |  |  |  |
| Yes | 71 (66%) | 29 (88%) | 8 (89%) | 34 (50%) |
| No | 3 (3%) | 0 | 0 | 3 (44%) |
| Not mentioned | 36 | - | - |  |
| **Total treatment days (mean days)** | 35±20 | 43±44 | 19±13 | 32±23 |
| **Prognosis** |  |  |  |  |
| Cure | 37 (34%) | 13 (39%) | 3 (33%) | 21 (31%) |
| Turn for the better | 41 (37%) | 16 (48%) | 5 (56%) | 20 (29%) |
| Invalid | 1 (1%) | 0 | 0 | 1 (1%) |
| Death | 23 (21%) | 2 (6%) | 1 (11%) | 20 (29%) |
| Not mentioned | 8 | - | - | - |
| **Systemic glucocorticoid therapy cured group -- mean days of treatment** | 36.10±20.53 | 43.80±23.28 | 20±13.23 | 34.5±18.78 |
| **Systemic glucocorticoid treatment cure group -- mean cumulative systemic glucocorticoid dosage (mg, in prednisone)** | 2389±1447 | 2639±2035 | 1200±990 | 2414±1100 |
| **Systemic glucocorticoid + immunoglobulin therapy/death** |  |  |  |  |
| Apply | 57/14 (25%) | 8/1 (13%) | 5/1 (20%) | 44/12 (27%) |
| Not Applied | 44/5 (11%) | 21/1 (5%) | 3/0 | 20/4 (20%) |
| **Systemic glucocorticoid + immunoglobulin therapy/cure** |  |  |  |  |
| Apply | 57/17 (30%) | 8/3 (38%) | 5/1 (20%) | 44/13 (30%) |
| Not Applied | 44/19 (43%) | 21/9 (43%) | 3/2 (67%) | 20/8 (40%) |
| **Systemic glucocorticoid + immunoglobulin treatment cured group -- average treatment days** |  |  |  |  |
| Apply | 35.73±20.75 | 59.67±30.01 | 5 | 32.00±11.87 |
| Not Applied | 36.44±20.99 | 37.00±18.24 | 27.50±3.53 | 38.43±27.12 |
| **Systemic glucocorticoid + immunoglobulin treatment cured group -- average cumulative glucocorticoid dosage (mg, in prednisone)** |  |  |  |  |
| Apply | 2727.48±1678.36 | 5500.00±707.11 | 500 | 2395.70±1148.25 |
| Not Applied | 2021.50±1101.05 | 1684.67±1154.52 | 1900 | 2450.00±1124.41 |
| **Systemic glucocorticoid + biologics for treatment/death** |  |  |  |  |
| Apply | 11/2 (18%) | 3/0 | 0 | 8/2 (25%) |
| Not Applied | 90/17 (19%) | 26/2(8%) | 8/1 (13%) | 56/14 (25%) |
| **Systemic glucocorticoid + biologics for treatment/cure** |  |  |  |  |
| Apply | 11/5 (45%) | 3/2 (67%) | 0 | 8/3 (38%) |
| Not Applied | 90/31 (34%) | 26/10 (38%) | 8/3 (38%) | 56/18 (32%) |
| **Systemic glucocorticoid + biologics treatment cure group -- average treatment days** |  |  |  |  |
| Apply | 42.75±32.10 | 62.50±38.89 | - | 23.00±4.24 |
| Not Applied | 35.11±18.96 | 39.13±18.86 | 20.00±13.23 | 35.94±19.46 |
| **Systemic glucocorticoid + biologics treatment cure group -- average cumulative systemic glucocorticoid dosage (mg, in prednisone)** |  |  |  |  |
| Apply | 2823.75±2027.34 | 3022.50±2796.61 | - | 2625.00±2085.97 |
| Not Applied | 2305.71±1360.4 | 2510.50±2038.90 | 1200±989.95 | 2381.31±1019.77 |
| **Systemic glucocorticoid + plasma therapy/death** |  |  |  |  |
| Apply | 9/2 (22%) | 0 | 2/0 | 7/2 (29%) |
| Not Applied | 92/17 (18%) | 29/2(7%) | 6/1 (17%) | 57/14 (25%) |
| **Systemic glucocorticoid + plasma therapy/cure** |  |  |  |  |
| Apply | 9/3 (33%) | 0 | 2/1 (50%) | 7/2 (29%) |
| Not Applied | 92/33 (36%) | 29/12 (41%) | 6/2 (33%) | 57/19 (33%) |
| **Systemic glucocorticoid + plasma therapy cured group -- mean days of treatment** |  |  |  |  |
| Apply | 27.33±4.04 | - | 25 | 28.50±4.95 |
| Not Applied | 37.04±21.39 | 43.80±23.28 | 17.50±17.68 | 35.25±19.82 |
| **Systemic glucocorticoid + plasma treatment cure group -- mean cumulative systemic glucocorticoid dosage (mg, in prednisone)** |  |  |  |  |
| Apply | 2138.33±237.50 | - | 1900 | 2257.50±166.17 |
| Not Applied | 2422.73±1541.82 | 2638.50±2035.39 | 500 | 2437.85±1184.95 |
| **Systemic glucocorticoid + antibiotic treatment/death** |  |  |  |  |
| Apply | 32/5 (16%) | 4/0 | 5/1(20%) | 23/4 (17%) |
| Not Applied | 69/14 (20%) | 25/2(8%) | 3/0 | 41/12 (29%) |
| **Systemic glucocorticoid + antibiotic treatment/cure** |  |  |  |  |
| Apply | 32/13 (41%) | 4/1(25%) | 5/1 (20%) | 23/11 (48%) |
| Not Applied | 69/23 (33%) | 25/11(44%) | 3/2 (67%) | 41/10 (24%) |
| **Systemic glucocorticoid + antibiotic treatment cured group -- average treatment days** |  |  |  |  |
| Apply | 33.78±26.23 | - | 5 | 37.38±25.56 |
| Not Applied | 37.05±18.35 | 43.80±23.28 | 27.50±3.54 | 32.20±12.04 |
| **Systemic glucocorticoid + antibiotic treatment cure group -- average cumulative systemic glucocorticoid dosage (mg, in prednisone)** |  |  |  |  |
| Apply | 2353.13±1287.89 | - | 500 | 2617.86±1131.81 |
| Not Applied | 2405.29±1553.67 | 2638.50±2035.39 | 1900 | 2235.25±1144.90 |

^a^: Includes nasopharynx, bile duct, gallbladder, ear, groin, testis, cervix, mouth, urothelium, prostate, kidney, esophagogastric junction, scalp, thymus, eyes, limbs

^b^:Includes cholangiocarcinoma, mesothelioma, non-keratinous carcinoma, adenocarcinoma/non-small cell carcinoma, not specified
