# Supplementary material for: Clinical characteristics of Stevens-Johnson syndrome/toxic epidermal necrolysis-like reactions induced by immune checkpoint inhibitors
Source: Oncologist. 2025 Jun 10;30(6):oyaf143. doi: 10.1093/oncolo/oyaf143 (PMC12149091; doi:10.1093/oncolo/oyaf143)
Supplement: oyaf143_suppl_Supplementary_Tables_S2 [file oyaf143_suppl_supplementary_tables_s2.docx]

**Supplemental Table S2：Summary of extensive epidermal necrosis response stratified according to results**

| **SJS/TEN and TEN****-like irAEs** | n（%） | |
| --- | --- | --- |
|  | **Improvement/cure group**   **Death group** | |
| **Total** | 49cases | 21cases |
| **Gender** |  |  |
| Male | 35 (71%) | 12 (57%) |
| Female | 14 (29%) | 9 (43%) |
| **Severity** |  |  |
| SJS/TEN-like | 8 (16%) | 1 (5%) |
| TEN-like | 41 (84%) | 20 (95%) |
| **Tumor site，n（%）** |  |  |
| Lung | 15 (31%) | 3 (14%) |
| Liver | 5 (10%) | 3 (14%) |
| Gastrointestinal tract | 11 (22%) | 3 (14%) |
| Others | 15 (31%) | 2 (10%) |
| Unmarked | 3 (6%) | 10 (48%) |
| **Tumor type，n（%）** |  |  |
| Squamous carcinoma | 13 (27%) | 3 (14%) |
| Melanoma | 7 (14%) | 10 (48%) |
| Adenocarcinoma | 8 (16%) | 2 (10%) |
| Non-small cell carcinoma | 3 (6%) | 1 (5%) |
| Others | 18 (37%) | 5 (24%) |
| **Tumor discovery - Application of ICIs duration (mean month)** | 21.25±42.52 | 24.8±35.88 |
| **ICIs usage, n (%)** |  |  |
| PD-1 inhibitors and PD-L1 inhibitors ^a^ | 46 (94%) | 15 (71%) |
| Others: PD-1+CTLA-4 inhibitors (all ipilimumab + nivolumab) or not specified | 3 (6%) | 6 (29%) |
| **Incubation period (mean days)** | 55.77±102.47 | 30.31±30.31 |
| **Involves special parts such as eyes, lips, etc., n (%)** |  |  |
| Yes | 30 (91%) | 8 (100%) |
| No | 3 (9%) | 0 |
| Not mentioned | 16 | 13 |
| **Treatment with systemic glucocorticoids, n (%)** |  |  |
| Apply | 48(98%) | 17 (81%) |
| Not Applied | 1 (2%) | 4 (19%) |
| Average treatment days | 32.12±18.41 | 34±32.52 |
| Mean cumulative systemic glucocorticoid dose (mg, in prednisone) | 2247.92±1578.05 | 2526.25±2018.43 |
| **Systemic glucocorticoid + immunoglobulin therapy, n (%)** |  |  |
| Apply | 32 (67%) | 13 (62%) |
| Not Applied | 16 (33%) | 8 (38%) |
| Average treatment days | 30.67±14.24 | 35.3±35.30 |
| Mean cumulative systemic glucocorticoid dose (mg, in prednisone) | 1938.56±1250.69 | 2526.5±2120.36 |
| **Systemic glucocorticoid + antibiotic therapy, n (%)** |  |  |
| Apply | 22 (46%) | 5 (24%) |
| Not Applied | 26 (54%) | 16 (76%) |
| Average treatment days | 34.63±24.89 | 50±47.61 |
| **Total treatment days (mean days)** | 31.48±18.66 | 32±31.96 |
| **Total mean cumulative systemic glucocorticoid dose (mg, in prednisone)** | 2247.92±1578.05 | 2526.25±2018.43 |

^a^: There was only one death with PD-L1 inhibitors (atezolizumab)
